# Intergenerational Transmission of Traumatic Stress and Resilience Among Cambodian Immigrant Families Along Coastal Alabama: Family Narratives

**DOI:** 10.1089/heq.2020.0142

**Published:** 2021-06-18

**Authors:** Chansophal Mak, Denise C. Lewis, Desiree M. Seponski

**Affiliations:** ^1^ Marriage and Family Therapy, Department of Human Development and Family Science, University of Georgia, Athens, Georgia, USA.; ^2^ Human Development and Family Science, Department of Human Development and Family Science, University of Georgia, Athens, Georgia, USA.

**Keywords:** traumatic stress, resilience, Cambodian immigrant families, intergeneration, ecological social determinants of health

## Abstract

**Purpose:** Intergenerational transmission of psychological trauma is an ongoing global public health concern. Cambodia experienced ∼4 years of genocide, causing about 2 million deaths. Many survivors fled and resettled in the United States where they continued to face the psychological and relational consequences of forced displacement, with limited access to mental health treatment. This study employed an ecological social determinants of health framework to explore how resettled families discussed traumatic experiences and resilience transmitted across three generations.

**Methods:** Narrative inquiry-guided, in-depth semistructured interviews were conducted with intergenerational families: five grandparents, six parents, and nine adult children. The interview protocol included developing detailed family genograms that facilitated the sharing of experiences of living through the Cambodian genocide (1975–1979) and resettlement to the United States in the 1980s. A thematic data analysis was conducted across individual and family experiences before, during, and after the genocide and resettlement.

**Results:** The findings highlight parent–child relationships as the primary mechanism of intergenerational transmission of traumatic stress and resilience among Cambodian immigrant families. Specifically, high parental expectations, authoritarian parenting, corporal punishment, and submissive communication styles were reported. On the other hand, strong bonds and less hierarchy between parents and children were found to be resilience factors among this population.

**Conclusions:** The results of this qualitative study underscore the need for a systemic mental health conceptualization for practitioners working with resettled Cambodian families to overcome the cycle of intergenerational transmission of traumatic stress and promote resilience postresettlement.

## Introduction

Traumatic stress is a global public health concern.^1^ Traumatic events such as civil wars and genocides have caused death and the destruction of family structures across the globe,^2,3^ impacting the generations exposed and subsequent generations left to deal with the consequent displacement and resettlement.^2,4–6^ Exposure to traumatic events and forced displacement impact one's entire ecology with long-term intergenerational consequences.^2,7^

Almost 2 million Cambodians died during the 1975–1979 genocide^8,9^ and about 15,8000 survivors immigrated to the United States.^9,10^ Cambodian immigrant families' experiences of the genocide, clear and ambiguous losses, family separation, forced displacement, and refugee immigrant status in the United States put them at risk for mental health disparities.^2,7,11,12^ Yet, over a period of 40 years, only one study was published on intergenerational transmission of traumatic stress among Cambodians, examining parenting styles.^13^ A number of studies conducted with war-affected populations, such as Holocaust survivors, former child soldiers, and Ukrainian genocide survivors, pointed out that parent–child relationships in the forms of parenting practices, parents' projection of their own needs and fear-based emotions, and communication patterns composed the main mechanisms of intergenerational transmission of traumatic stress and resilience.^4,14,15^ However, to date, no research has investigated intergenerational transmission of traumatic stress among Cambodian immigrants even though three generations of genocide survivors live largely unseen and unheard in their resettlement countries. This exploratory study aims to understand and begin to document mechanisms of intergenerational transmission of traumatic stress among Cambodian immigrant families. We employed an ecological social determinants of health model to frame the study, focusing on societal factors such as social and economic factors that are linked to mental and physical health outcomes.^12^

### Transmission of traumatic stress

A holistic conceptualization of traumatic stress that includes biological factors, developmental history, family systems, and social context is crucial to fully comprehend this phenomenon.^16^ Previous studies have shown the main mechanisms of intergenerational transmission of traumatic stress passing through parent–child interactions. Specifically, a study investigated secondary trauma among Holocaust survivors and revealed that the transmission of traumatic stress to the third generation occurred mainly as a result of living in the community with war history.^17^ Long-term effects of traumatic stress among second and third generations were studied across three groups: Group 1 (third generation with both parents who are Holocaust survivors), Group 2 (third generation with one parent who is a Holocaust survivor), and Group 3 (third generation with parents who are not Holocaust survivors). Only Group 1 demonstrated evidence of trauma symptoms long after first-generation exposure. Similarly, emotional reactions of traumatic stress were identified in a study among Brazilian Holocaust survivor offspring to determine how trauma passed from parents to children through their communication patterns.^14^ The parents communicated fear, avoidance, guilt, feelings of helplessness, and submission to their offspring. These studies emphasize intergenerational transmission of traumatic stress with important implications for family dynamics and the well-being of future generations.

Intergenerational transmission of traumatic stress through the parent–child relationship was also found among Ukrainian genocide survivors and Burundian former child soldiers.^4,15^ These survivors demonstrated a wide range of fear-related emotions (i.e., sadness, anxiety, and anger) and fear-related coping strategies (i.e., stockpiling and food consumption) passed to subsequent generations over eight decades.^4^ Parenting practices (i.e., the use of corporal punishment and showing aggression) also caused aggressive behaviors among their offspring.^15^ Traumatic stress is sustained in affected populations for prolonged periods of time due to its psychological and relational consequences and the common lack of resources in the community. There is an ongoing need to understand trauma transmission processes and related social determinants of risk and protective factors; hence, its transgenerational effects can be disrupted.

### Resilience and traumatic stress

Resilience is the ability of an individual or family to use its internal resources (i.e., strong family bond and loyalty) to enhance physical and relational health to cope, adapt to, and bounce back from adversities.^18,19^ Resilience plays an important role in the experience of war-affected and displaced populations.^18^ Most importantly, achieving resilience is highly dependent on the level of support available in the community and host countries.^20^

A study among Brazilian Holocaust survivor offspring revealed a capacity to bounce back from adversities.^14^ Similarly, another study identified transmission of resilience from the second to the third generation among Holocaust survivors, referring to protective factors such as a middle-class social–economic status and higher educational attainment of the parents.^21^ A study among former child soldiers revealed resilience in the form of responsibility taking.^15^ The first generation taught sons to take responsibility in the family and daughters to marry responsible men.

The overarching framework for this study allows interventionists and mental health professionals to trace intergenerational transmission of trauma and resilience among trauma-affected populations by incorporating an emic understanding of their culture, history, and lived experiences.^22–24^ The transgenerational trauma and resilience genogram framework used in this study integrates trauma theory, ecological system theory, resilience, cultural responsiveness, and a social justice lens to view traumatic experiences across generations.^25^ We included the use of genograms to aid in the examination of transmission of trauma and resilience across three generations of Cambodian immigrant families. Families were interviewed using narrative inquiry principles and genograms focusing on significant periods of time before, during, and after the genocide and resettlement. Interviews aimed to answer the main research question: What are the mechanisms of intergenerational transmission of traumatic stress and resilience among Cambodian immigrant families?

## Methods

This study was conducted in a predominantly rural Cambodian community along coastal Alabama. Purposive and snowball sampling methods were used to recruit participants. Narrative inquiry principles enabled researchers to capture the depth and flow of participants' experiences^26,27^ and allowed a deep understanding of participants' positioning in relation to multiple social locations (e.g., gender roles, social–economic status, and immigrant status).^28^ Table 1 illustrates demographic information of 20 participants from six families.

**Table 1.** Demographic Information of Participants

Family/generation	Gender	Age	Ethnicity	Education
1. F1/G1	Female	56	Kampuchea Krom	No
2. F1/G2	Female	37	Kampuchea Krom	Dropped out
3. F1/G3	Male	19	Kampuchea Krom	High school
4. F2/G1	Female	63	Chinese Cambodian	No
5. F2/G2	Female	40	Chinese Cambodian	Dropped out
6. F2/G3	Male	18	Chinese Cambodian	First-year college
7. F3/G1	Female	79	Cambodian	No
8. F3/G2	Female	58	Cambodian	No
9. F3/G3	Female	33	Cambodian	Associate degree
10. F3/G3	Female	26	Cambodian	High school
11. F3/G3	Female	23	Cambodian	College
12. F4/G1	Male	78	Cambodian	No
13. F4/G2	Male	55	Cambodian	Dropped out
14. F4/G3	Female	31	Cambodian	Dropped out
15. F5/G1	Female	71	Cambodian	No
16. F5/G2	Male	51	Cambodian	College
17. F5/G3	Male	18	Cambodian	High school
18. F6/G2	Female	46	Kampuchea Krom	Dropped out
19. F6/G3	Male	28	Kampuchea Krom	High school
20. F6/G3	Female	18	Kampuchea Krom	High school

F1/G1, Family 1 Generation 1; F1/G2, Family 1 Generation 2; and F1/G3, Family 1 Generation 3. F2/G1, Family 2 Generation 1; F2/G2, Family 2 Generation 2; and F2/G3, Family 2 Generation 3. F3/G1, Family 3 Generation 1; F3/G2, Family 3 Generation 2; and F3/G3, Family 3 Generation 3. F4/G1, Family 4 Generation 1; F4/G2, Family 4 Generation 2; and F4/G3, Family 4 Generation 3. F5/G1, Family 5 Generation 1; F5/G2, Family 5 Generation 2; and F5/G3, Family 5 Generation 3. F6/G1, Family 6 Generation 1; F6/G2, Family 6 Generation 2; and F6/G3, Family 6 Generation 3.

After Institutional Review Board approval from a major university in the South, Cambodian community leaders were enlisted to aid in participant recruitment. Community members who indicated interest in the study were asked for verbal and written consent. The interviews, transcription, and open coding were conducted in the original languages (i.e., Khmer or English) by the first author who is Cambodian and fluent in both languages. In-depth semistructured interviews were conducted by following specific questions (e.g., What is your experience of the genocide and migration? How have these experiences impacted you and your family? Tell me about how you do parenting? How is it similar or different from how you were parented?) to track mechanisms of trauma and resilience.^29^ To minimize potential psychological risk, participants were asked for ongoing consent, debriefed at the interview conclusion, and informed about the possibility of a local mental health referral.

To ensure the accuracy of the transcripts, the primary researcher transcribed and confirmed that the written scripts and the audio file matched. Audio interviews, field notes, memos, and genograms compose the data set. Data were triangulated and member-checked by the three authors to enhance trustworthiness and richness of participants' stories.^30–32^ Inductive, deductive, and abductive approaches along with two cycles of coding were performed.^33^ Narrative thinking and categorical thinking were used to identify codes and themes.^27,34,35^ After data were open coded in their original languages, Khmer codes were translated into English. Codes were then arranged chronologically into time plots representing before, during, and after the genocide to capture how a group of codes and themes changed over time.^31,32^ Analytic memos, field notes, and genograms of each family also informed identification of codes and themes.^36^

## Results

Each generation spoke about their experiences associated with the genocide. Table 2 is a full elaboration of the thematic analysis in this study. However, for the purposes of this article, only themes directly associated with intergenerational transmission of trauma and resilience in parent–child relationships are described. Findings are elaborated below within two categories comprising themes and accompanying quotes labeled by each respective family member and generation.

**Table 2.** Main Themes

Family/generation	Main themes	
1. F1/G1 2. F1/G2 3. F1/G3	**1. Submissive parent–child relationship** **2. Mixed parenting practices** **3. Filial piety** 4. Starting a new life	5. Loss 6. Displacements 7. Coping strategies 8. Fear of reoccurrence
4. F2/G1 5. F2/G2 6. F2/G3	**1. Submissive parent–child relationship** **2. Mixed parenting practices** **3. Filial piety** 4. Coping strategies	5. Loss 6. Displacements 7. Starting a new life 8. Fear of reoccurrence
7. F3/G1 8. F3/G2 9. F3/G3 10. F3/G3 11. F3/G3	**1**. **Submissive parent–child relationship** **2. Mixed parenting practices** **3. Filial piety** 4. Marital relationship 5. Coping strategies 6. Child labor	7. Displacements 8. Education 9. Fear of reoccurrence 10. Loss 11. Starting a new life
12. F4/G1 13. F4/G2 14. F4/G3	1**. Submissive parent–child relationship** **2. Mixed parenting practices** **3. Filial piety** 4. Leadership/business 5. Coping strategies 6. Starting a new life	7. Child labor 8. Marital relationship 9. Loss 10. Fear of reoccurrence 11. Displacements
15. F5/G1 16. F5/G2 17. F5/G3	1. **Submissive parent–child relationship** **2. Mixed parenting practices** **3. Filial piety** 4. Conflicting marital relationship 5. Coping strategies 6. Loss	7. Displacements 8. Starting a new life 9. Education 10. Child labor 11. Fear of reoccurrence
18. F6/G2 19. F6/G3 20. F6/G3	**1. Submissive parent–child relationship** **2. Mixed parenting practices** **3. Filial piety** 4. Conflicting marital relationship 5. Starting a new life	6. Loss 7. Displacements 8. Coping strategies 9. Education

Only the themes in bold letters are included in this study since they are related to parent–child relationships and intergenerational transmission of traumatic stress and resilience.

### Category I: Intergenerational transmission of traumatic stress

#### Theme I: Submissive parent–child relationships

The theme of submissive parent–child relationships resulting from trauma exposure occurs across all three generations. G1 described their adherence to a submissive communication in parent–child relationships during their time in Cambodia. Submissive communication refers to unquestioning acquiescence to parental wishes and guidance and not expressing their own needs.

I listened to my parents. They gave me birth… [have to listen to them]. I dared not do anything as I wish….[checking if they are happy] (F4/G1).

Submissive communication remains a common style among many Cambodian immigrant children.

I want to become a teacher, but my dad [disagreed]… Instead I will study pharmacy or medicine (F5/G3).

My mum asked me to come over here to take care of my [sick] grandma… I've been here for about 7 months [taking care of her] (F3/G3) (aged 23 years).

Still others engage in a more passive pattern of listening, but not always following their parents' wishes.

My mother was very strict [controlling]… I am not allowed to have a boyfriend [against Cambodian cultural value] (F3/G3) (aged 33 years).

The theme of submissive parent–child relationships was also seen in descriptions of life during the genocide by both G1 and G2 participants.

During the genocide, my parents decided for me to get married. [arranged marriage]… [protection from sexual abuse] from Pol Pot people (F2/G1).

Both generations described authoritarian parenting as the most common style in Cambodia. This pattern remains strong in families in the United States, particularly among G1 Cambodian immigrants, whereby children have little freedom in their decision-making.

#### Theme II: Mixed parenting practices after the genocide and resettlement (generations 1, 2, and 3)

Both G1 and G2 Cambodian immigrant parents use authoritarian parenting and report giving orders to their children to follow their advice. They reported that children often practice submissive communication with their parents and older family members. Some G2 parents practice corporal punishment, but they are also aware of child protection laws in the United States. A participant shared: “I love and listen to my parents. I also want my children to listen to me, but I don't think they do. They are American kids and we can't touch them” (F3/G2). When children do not adhere to their parents' wishes, some parents become angry and resort to corporal punishment. Another participant described her husband as “violent and cruel” in his use of corporal punishment of her and their children.

My second husband was very violent [domestic violence]…[corporal punishment] to my and our daughters (F3/G1).

Strong parental orders, limited freedom in decision-making, and the use of corporal punishment continued after resettlement in the United States.

G3 participants reported using a combination of authoritarian and permissive parenting styles. A G3 participant described feeling close to their children and offering them freedom.

I feel so close to my children [Giving freedom of choice]… [No hierarchy] with my children (F3/G3) (aged 33 years).

Conversely, participant F3/G3 (aged 26 years) reported that her and her parents' parenting styles are similar, using corporal punishment and, at times, the more consistent pattern of time out used in the United States.

I think my parenting is similar to my parents' [using stick to discipline]…. [using time out] works with my small kids (F3/G3) (aged 26 years).

These two parenting narratives reveal that participants both follow intergenerational strategies and change over time to reflect patterns more closely associated with U.S. parenting styles.

### Category II: Intergenerational transmission of resilience

Narratives from each generation demonstrate intergenerational transmission of resilience. Particularly, filial piety, a core concept in Cambodian families' intergenerational relationships, refers to the strong family bond and the roles of offspring to repay their parents and older generations.^37^

#### Theme I: Filial piety within parent–child relationships

Both G1 and G2 participants were accustomed to following tenants of filial piety in their lives through helping with housework and in cultivating rice for the family's consumption.

Before the genocide, I had to help my parents at the rice field. [child labor]… [no chance to study] (F3/G1).

During the genocide, filial piety remained a core concept despite life-threatening events. Some G2 participants reported taking risks stealing food for their family, while others reported listening to their parents' orders due to their trust in them.

My father knows everything [our family hero]. He saved us and his parents from death during the genocide (F4/G2).

I was assigned to work in the rice field… [stealing food]. I could have been killed had I got caught… [taking risk for my family] (F3/G2).

G2 and G3 families reported that intergenerational transmission of filial piety continued as a strong guide in parent–child relationships. Many continued to engage as caregivers to older generations. Filial piety among this population did not significantly change over time.

An additional continuation of filial piety is the adherence to expectations of firstborn children to take on the roles of the parents when the parents are not present or when they are incapable of performing their roles. This places the decision-making role on the firstborn.

My father was at the emergency room [surgery for brain tumor]… he also has lung cancer. As the firstborn child [deciding for the family]… (F3/G2)

I am a firstborn child. [being role model] to my siblings… I can't mess up…[getting college admission] (F2/G3).

Resilient parent–child relationships are reflected in the desire of older generations to provide education for younger generations. Even though G1 participants did not have the opportunity to study due to massive disruption to their lives in Cambodia, they recognized the value of education for younger generations. One participant expressed pride that her son was the first to finish college shortly after their arrival in the United States.

My son liked to study [diligent and hardworking]… Now he is very successful in his career as an accountant (F5/G1).

Multiple narratives revealed ways in which families adhere to filial piety and enhance their family's resilience after experiencing multiple traumas associated with genocide and resettlement.

## Discussion

The discussion is organized by situating study results within an ecological social determinants of health framework that links intergenerational transmission of traumatic stress and resilience among Cambodian immigrant families to existing literature. The discussion also elaborates on implications for practitioners and researchers working with Cambodian immigrant families across multiple generations.

High parental expectations and projections of their needs force children to follow and respond to their parents' needs, while ignoring their own needs. Cambodian immigrant parents projected unresolved needs and expectations through fear-related emotions (i.e., sadness, anxiety, and anger) to their children. These fear-based parent–child relationships create blurring of boundaries that can be described as identity confusion often linked to crisis responses arising in the children.^38^

Corporal punishment and authoritarian parenting were common practices in Cambodia and often continued in the United States, although reportedly at reduced intensity. Both are signs of an inability to control one's anger and frustration and have been shown to harm children.^39,40^ Corporal punishment forces children to comply immediately, but leads to children's aggression^15,41^ such as anger outbursts, yelling, stamping, and domestic violence. G3 participants did not experience living through the genocide; however, they manifested trauma responses such as those present in their parents' relational and communication styles. Our findings parallel previous findings of indirect exposure of Holocaust survivors: the third generation did not live through the genocide, yet they still manifest trauma responses such as those in their parents through parent–child relationships.^42,43^

Regarding intergenerational resilience, a strong bond in parent–child relationships refers to a sense of belonging, a sense of togetherness, and a sense of hope and meaning in life.^44–46^ This strong bond in parent–child relationships acts as a protective factor against life adversities. Particularly, G3 Cambodian immigrants reported that they feel close and give freedom to their children. This generation also shared that they spend their free time in leisurely activities, while the second generation could not do that when they were younger because of poverty and limited resources, which resettlement countries often fail to offer to war-affected populations who experienced and accumulated traumatic stress, including acculturation stress, during their displacement (i.e., pre-, during, and postmigration).^2,7^ Most importantly, acculturation stress was also found to be a social determinant of mental health among migrants.^47^ Because the G1 and G2 generations had to ensure the basic needs of their families, they had no chance to engage in leisure activities as G3. The responsibilities and caring for older generations toward subsequent generations create strong bonding in parent–child relationships and increased family resilience.^18^ This aligns with the findings of intergenerational trauma among Holocaust survivors, emphasizing the importance of healthy bonding in parent–child relationships as a protective factor against trauma transmission.^46^

Several studies pointed to examples of intergenerational family resilience reported by the generations that directly exposed to traumatic events.^14,15,18,21^ Successful resettlement requires comprehensive structural resources and support from the resettlement countries for war-affected populations so that they can address their chronic traumatic stress, which hinders their ability to develop family resilience and perform healthy parenting.^2,7,19,20^ Cambodian immigrants who suffer from genocide traumatic stress, loss of family members, displacement to foreign countries due to fear of safety, and severe poverty manage to provide very limited amount of warmth and care to their families unless they have more access to support systems in the resettled country to address mental and relational health concerns after their resettlement. The individual mental health, parenting, and family relationships of these communities will continue to be compromised unless the social–political landscape in the United States changes to more adequately address the needs of these families. Considering the ecological determinants of health framework,^12^ resettlement families should be also screened for mental health in combination with physical health, and school staff should be trained to recognize and support children and parents affected by war, trauma, and resettlement.

## Conclusions

Parent–child relationships drive transmission of traumatic stress and resilience across generations and can be the antecedent of communication styles and coping strategies. Older Cambodian immigrants continue to pass their trauma responses to their descendants, often unaware of unhealthy parent–child patterns. At other times, a strong parent–child bond protects Cambodian immigrant offspring from the effects of intergenerational trauma. With proper support in the resettlement country, these families would have the opportunity to address their individual trauma symptoms and disrupt their intergenerational effects.

Two important clinical implications arise from this study: (1) proper psychological and relational assessments and culturally responsive parenting interventions and (2) trauma healing resources that address needs of individuals and their families are needed within resettlement communities. Clinicians, practitioners, and policy makers need to pay attention to parent–child relationships, identifying stressors and unmet needs for individual parents and the resulting stress patterns, communication styles, discipline methods, and emotional distress they pass on to children. Supporting parents directly impacts the children, and both generations can better meet their needs. To achieve individual and relational mental health, the resettlement country needs to involve and offer enough support to war-affected populations. Because of the traumatic stress experienced by G1 and G2 pre- and during migration, as well as the acculturation stress experienced by all three generations postmigration, culturally responsive and effective systemic and multilevel interventions are required (i.e., individual, family, and community) to disrupt the intergenerational traumatic stress among this population.
